# Dcsbis (PA2771) from *Pseudomonas aeruginosa* is a highly active diguanylate cyclase with unique activity regulation

**DOI:** 10.1038/srep29499

**Published:** 2016-07-08

**Authors:** Ying Chen, Shiheng Liu, Cuilan Liu, Yan Huang, Kaikai Chi, Tiantian Su, Deyu Zhu, Jin Peng, Zhijie Xia, Jing He, Sujuan Xu, Wei Hu, Lichuan Gu

**Affiliations:** 1 State Key Laboratory of Microbial Technology, School of Life Sciences, Shandong University, Jinan, Shandong, 250100, China; 2 Institute for metabolic and neuropsychiatric disorders, Binzhou Medical University, Binzhou, Shandong, 256600, China; 3 College of Life Sciences, Shandong Normal University, Jinan, Shandong, 250014, China

## Abstract

C-di-GMP (3’,5’ -Cyclic diguanylic acid) is an important second messenger in bacteria that influences virulence, motility, biofilm formation, and cell division. The level of c-di-GMP in cells is controlled by diguanyl cyclases (DGCs) and phosphodiesterases (PDEs). Here, we report the biochemical functions and crystal structure of the potential diguanylase Dcsbis (PA2771, a diguanylate cyclase with a self-blocked I-site) from *Pseudomonas aeruginosa* PAO1. The full-length Dcsbis protein contains an N-terminal GAF domain and a C-terminal GGDEF domain. We showed that Dcsbis tightly coordinates cell motility without markedly affecting biofilm formation and is a diguanylate cyclase with a catalytic activity much higher than those of many other DGCs. Unexpectedly, we found that a peptide loop (protecting loop) extending from the GAF domain occupies the conserved inhibition site, thereby largely relieving the product-inhibition effect. A large hydrophobic pocket was observed in the GAF domain, thus suggesting that an unknown upstream signaling molecule may bind to the GAF domain, moving the protecting loop from the I-site and thereby turning off the enzymatic activity.

C-di-GMP is a ubiquitous second messenger in bacteria. It was first reported as an activator regulating cellulose synthesis^1^,^2^. C-di-GMP modulates diverse cellular functions, such as motility, biofilm formation, virulence and cell division, by influencing gene transcription, signal transduction, protein secretion and stability, and enzymatic activity^3^,^4^,^5^. The level of c-di-GMP is controlled by the coordination of two classes of enzymes, diguanyl cyclases (DGCs) and phosphodiesterases (PDEs)^6^. The DGCs catalyze the synthesis of c-di-GMP from GTPs and contain the conserved amino acid sequence motif “GGDEF” (or “GGEEF”). PDEs hydrolyze c-di-GMP to linear pGpG and GMP and normally contain an EAL domain or an HD-GYP domain^7^,^8^,^9^,^10^,^11^,^12^,^13^. As the synthesizer of c-di-GMP, DGCs play important roles in regulating the biological behaviors of bacteria. Therefore, revealing the biochemical mechanism of DGCs might provide an avenue for controlling infections and the toxicity of pathogenic bacteria.

The GGDEF domain is responsible for the catalytic activity of DGCs. Previous studies on GGDEF domain-containing proteins (PleD, WspR and XCC4471) have proposed that the activity of DGCs requires the cooperative action of two GGDEF domains that individually bind one molecule of GTP^14^. The dimerization of GGDEF domains enables catalysis by bringing two GTP-bound active sites together. Most GGDEF domains are linked to other accessory domains that are located in the N-terminus and act as signaling domains^14^,^15^. For example, the PleD protein contains an N-terminal CheY-like domain, and the phosphorylation of the CheY-like domain alters the surface of the protein and promotes dimerization, which subsequently activates the GGDEF activity^16^,^17^. Similar activation exists in the catalytic mechanism of WspR^18^. In XCC4471 from *Xanthomonas campestris*, the HAMP and TM domains are essential for the dimerization of DGC domains^19^.

In addition to activation by oligomerization, the activity of GGDEF domains can also be regulated by product inhibition. More than 60% of GGDEF domains contain a conserved RxxD motif that is bound to the product c-di-GMP and that facilitates product feedback inhibition^14^. The RxxD motif has been suggested to be the allosteric product inhibition site (I-site). The DGC activities of both PleD and WspR are regulated by non-competitive product inhibition via the binding of c-di-GMP to the allosteric I-site^20^,^21^. XCC4471 lacks the typical RxxD motif; the complex formed by XCC4471 and c-di-GMP indicates a competitive inhibition model blocking the DGC activity^19^. Product inhibition is critical for cellular c-di-GMP homeostasis.

Although the structures of the previously studied GGDEF domain-containing proteins have provided a general model for the mechanism of oligomerized activation and product inhibition, exceptions do exist in some proteins, especially in those with various additional signaling domains. Our study was focused on the protein product of the *PA2771* gene from the opportunistic pathogen *Pseudomonas aeruginosa* PAO1, which is a major drug-resistant secondary infection source in hospitals. The observation that PA2771 shows the same distribution as the type III-delivered cytotoxin-encoding exoS^22^, as well as its involvement in drug response^23^, makes it an interesting target.

The PA2771 gene is located on the genomic island of strain PAO1, which has not been reported in strain PA14 and six other *P. aeruginosa* strains^22^. According to the domain prediction from the amino acid sequence, the protein contains an N-terminal GAF (c**G**MP-specific phosphodiesterase, **a**denylyl cyclases and **F**hlA) domain, which is a common signaling domain, and a C-terminal GGDEF domain. This domain organization indicates that PA2771 synthesizes c-di-GMP. However, little is known regarding its enzymatic activity and the regulation of its activity. In addition, there is little known about its effect on bacterial behaviors, such as biofilm formation and motility.

Here, we show that PA2771 controls cell motility without substantially affecting biofilm formation. *In vitro* activity assays show that PA2771 is a highly active diguanyl cyclase, a result consistent with its possession of a GGDEF domain. To study the mechanism of PA2771 catalysis and regulation, we also crystallized full-length PA2771 and the GGDEF domain of PA2771 in complex with c-di-GMP. The crystal structure of full-length PA2771 revealed the active site and product inhibition site of the enzyme. In the full-length structure, PA2771 forms dimers via the GAF domain. Intriguingly, the two monomers in the dimer block the I-site of each other with a loop (protecting loop) extending from the GAF domain. However, in the GGDEF domain/c-di-GMP complex structure, the GGDEF domain dimerizes via the two c-di-GMPs from the inhibition sites. Because I-site product inhibition self blockage is a special feature of PA2771, we named the protein Dcsbis (diguanylate cyclase with a self-blocked I-site). From the structural and biochemical data, we propose a potential regulatory model for the regulation of the DGC activity of Dcsbis.

## Results

### PA2771 controls bacterial motility without affecting biofilm formation in PAO1

To study the biological function of PA2771 in PAO1, we constructed a PA2771 deletion mutant (*ΔPA2771*) of PAO1 and compared its behavior with that of the wild type strain (WT). According to previous studies, GGEDF-domain containing proteins, such as SadC^24^, influence the mobility and biofilm formation ability of bacteria. Here, we tested the two phenotypes in both the WT and ΔPA2771 strains.

**Figure 1:**
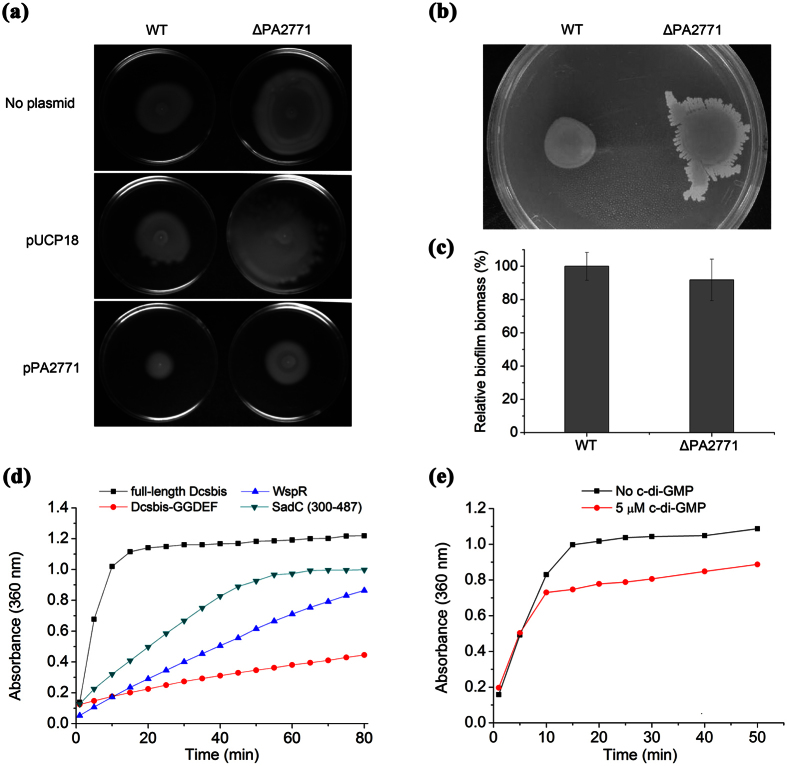
Motility/biofilm assay on the PA2771 deletion strain and the activity measurement of Dcsbis. (**a**) Swimming motility of WT PAO1 and PA2771 deletion strains (ΔPA2771). (**b**) Swarming motility of WT PAO1 and ΔPA2771 strains. (**c**) Biofilm assay of WT PAO1 and ΔPA2771 strains. (**d**) The DGC activity of full-length Dcsbis, Dcsbis-GGDEF, WspR, and SadC. (**e**) The DGC activity of full-length Dcsbis in the presence of 5 μM c-di-GMP.

Compared with the WT strain, the ΔPA2771 mutant showed a significantly increased ability to swim (Fig. 1a). Therefore, we hypothesized that the overexpression of Dcsbis would cause a deficiency in swimming motility. As expected, introducing a multi-copy plasmid carrying the PA2771 gene into the WT and ΔPA2771 strains significantly decreased the swimming motility (Fig. 1a). The ΔPA2771 mutant also displayed an altered swarming motility (Fig. 1b). Unexpectedly, the ability of the ΔPA2771 mutant to form a biofilm, compared with that of the WT strain, was not significantly different (Fig. 1c). This finding indicates that PA2771 has no detectable influence on biofilm formation.

### Dcsbis is a highly active diguanylate cyclase *in vitro*

According to sequence analysis, Dcsbis contains an N-terminal GAF domain and a C-terminal GGDEF domain. This analysis suggests that Dcsbis may possess DGC activity. Thus, we performed a DGC activity assay with full-length Dcsbis by using the pyrophosphate method^25^. The result showed that Dcsbis converts two GTPs into c-di-GMP and releases one molecule of pyrophosphate (PPi). To test whether the GGDEF domain is fully responsible for the DGC activity, we assayed the activity of the GGDEF domain (Residues 173 to 341). Interestingly, we found that the GGDEF domain of Dcsbis, compared with the full-length Dcsbis, exhibited only residual DGC activity (Fig. 1d). These results indicated that Dcsbis contains an active GGDEF domain but that the GAF domain is required to achieve full DGC activity.

To further assess the catalytic activity of Dcsbis, we compared its activity with the activity of the known DGCs WspR and SadC. DGC activity assays were performed for the three proteins at the same molar concentration, by following the previously mentioned procedure. Interestingly, Dcsbis showed a much higher DGC activity than WspR and SadC (Fig. 1d). This result raises an open question: how is the higher activity occurred in Dcsbis?

A sequence analysis indicated that the conventional product inhibition site (I site) of the RxxD motif also exists in Dcsbis, and it is located upstream of the GGEEF active site. The sequence is RPED (Arg-Pro-Glu-Asp). To investigate whether the I-site facilitates product inhibition by binding to c-di-GMP in Dcsbis, we explored the DGC activity of full-length Dcsbis in the presence of 5 μM c-di-GMP. The release of PPi was reduced in the presence of additional c-di-GMP, a result indicating that the production of new c-di-GMP was hindered (Fig. 1e). These results implied that the DGC activity of Dcsbis is also regulated by product inhibition. However, the product inhibition effect seems much weaker for Dcsbis compared with other studied DGCs.

### Crystal structures of Dcsbis

To investigate the molecular mechanism underlying the high activity of Dcsbis and the regulation mechanism of DGC activity by the N-terminal GAF domain, we determined the crystal structure of full-length Dcsbis. We first crystallized selenomethionine-incorporated Dcsbis. The structure was solved at a resolution of 2.9 Å by using the single-wavelength anomalous dispersion (SAD) technique. The higher resolution native structure (2.5 Å) was solved by molecular replacement, using one molecule from the selenomethionine structure as the search model. The details of the refinement data are shown in Table 1. Dcsbis was crystallized in the space group *P*2_1_ and contained four molecules in the asymmetric unit.

**Figure 2:**
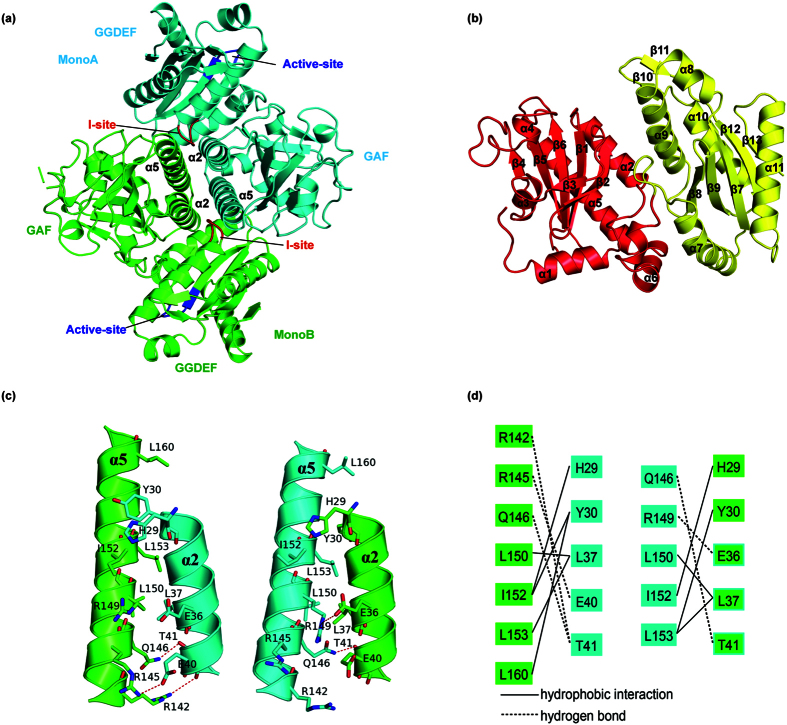
The structure of full-length Dcsbis. (**a**) The tightly associated homodimer of Dcsbis. The A-site and I-site of Dcsbis are colored in blue and red, respectively. (**b**) The overall structure of the Dcsbis monomer. The N-terminal GAF domain is colored in red, while the C-terminal GGDEF domain is colored in yellow. (**c**,**d**) The hydrogen bonds and hydrophobic interactions contributing to homodimerization.

The overall structure showed that the protein contains an N-terminal GAF domain and a C-terminal GGDEF domain, as predicted. The crystal packing analysis revealed the presence of a tightly associated homodimer, which was verified by gel filtration analysis (data not shown). The dimerization is mediated by helices α2 and α5 from the GAF domain of the two monomers (Fig. 2a,b).

A DALI server search^26^ against the Protein Data Bank failed to detect any known structures with significant similarities to full-length Dcsbis. This result suggested a novel arrangement between the GAF and GGDEF domains in Dcsbis. A DALI search with the GAF domain identified the most similar structure to be the GAF domain from *Acinetobacter sp*. phosphoenolpyruvate-protein phosphotransferase (PDB: 3CI6, RMSD 2.0 Å over 126 Cα atoms). However, the sequence identity was only 15%, which may imply differences in the function of the GAF domain in these two proteins, despite the similarity in their three dimensional folding.

Following the GAF domain, the GGDEF domain has a typical DGC structure. It superposes well with the GGDEF domain of the known DGC, PleD (with an RMSD of 2.3 Å for 162 Cα atoms) and WspR (with an RMSD of 2.3 Å for 162 Cα atoms). Similarly, Dcsbis contains a conserved active site in the GGEEF motif (A-site) and a conserved product inhibition site in the RxxD motif (I-site) located antipodal to the A-site. Interestingly, in the closely packed Dcsbis dimer, the A-sites of the two monomers face away from each other, and the I-site is buried in the structure (Fig. 2a). This result indicates that the native structure is a non-active mode of the protein.

**Figure 3:**
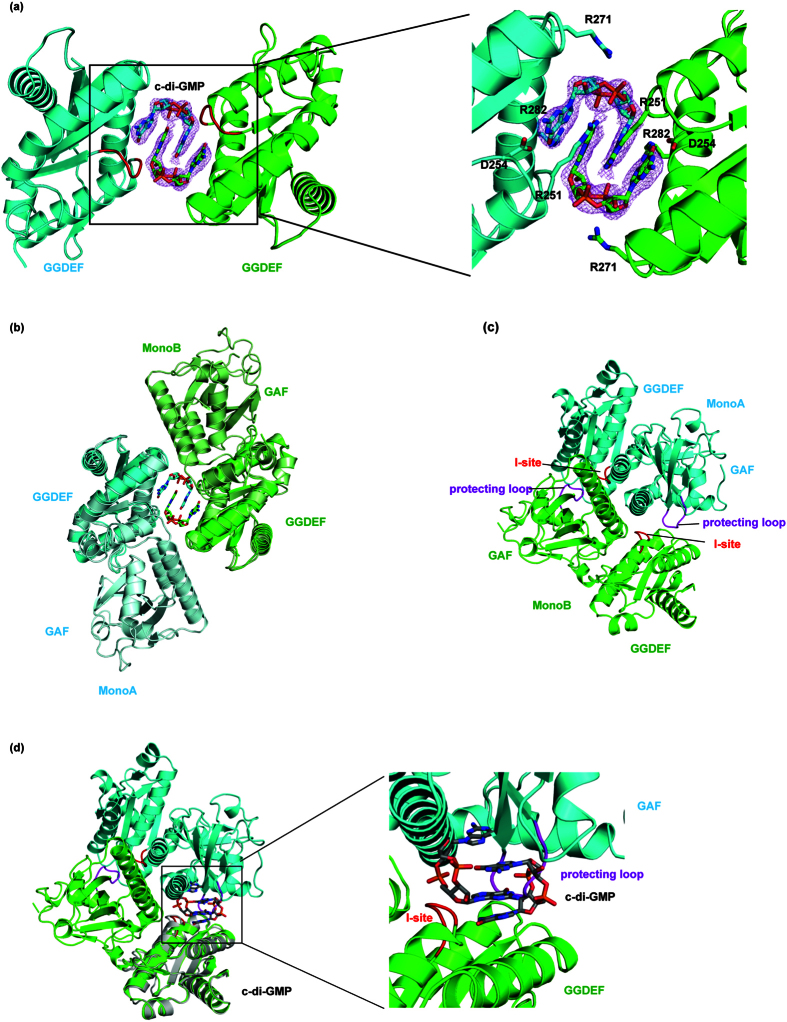
The structure of the GGDEF domain of Dcsbis in complex with c-di-GMP and the inhibition loop. (**a**) The dimer of the Dcsbis-GGDEF/c-di-GMP complex. The I-site and A-site of Dcsbis are in red and blue, respectively. (**b**) The superposition of the full-length Dcsbis monomer onto the Dcsbis-GGDEF/c-di-GMP complex dimer. The full-length protein is in lighter colors. (**c**) The peptide loop (protecting loop, residues 120–125) extending from the GAF domain blocks the inhibition site of Dcsbis. The protecting loop is colored in purple, whereas the I-site is red and the active site is blue. (**d**) The superposition of GGDEF/c-di-GMP complex onto the full-length structure of Dcsbis to model the position of c-di-GMP into the full-length Dcsbis structure. GGDEF/c-di-GMP are highlighted in gray. A close view of the c-di-GMP modeled into the Dcsbis structure.

To investigate how the GAF domain affects enzymatic function, we crystallized the GGDEF domain and solved the structure at 2.5 Å resolution in the *C*2 space group. The structure was determined using molecular replacement with the GGDEF domain from the full-length protein structure as the search model. There were two molecules in the asymmetric unit. The two monomers face each other, with the I-sites from both molecules in proximity. After molecular replacement, differences in electron density appeared between the I-sites of the two monomers. This extra density may be explained by building in two mutually intercalated c-di-GMP molecules (Fig. 3a). The final model of the Dcsbis-GGDEF structure was refined to an R_work_ of 20.14% and an R_free_ of 24.77%. The c-di-GMP dimer mediates the dimerization of the isolated GGDEF domains. A model of full-length Dcsbis in its inhibition state was generated by superposing the apo full-length structure onto the GGDEF dimer. The conformation accommodated the full-length protein without any clashes (Fig. 3b). Thus, dimerization via the c-di-GMP dimer may represent Dcsbis in its inhibited state.

Compared with the full-length structure, the structure of the GGDEF domain has no gross structural rearrangement, with an RMSD of 1.3 Å over 180 Cα atoms (Fig. 3b). Within the binding regions, Arg251, Asp254, Arg271 and Arg282 account for the main interactions with the dimeric c-di-GMPs (Fig. 3b). Importantly, Arg251 and Asp254 belong to the conserved product inhibition motif (RxxD).

### GAF domain of Dcsbis

GAF domains are one of the most widespread domains, and they exhibit diverse functions, including mediating the binding of small molecules (such as cAMP and cGMP) and protein dimerization^27^. To date, most ligands that recognize the GAF domain have not been discovered. The GAF domain of Dcsbis contains a six-stranded antiparallel β-sheet sandwiched by five α-helices (Fig. 2b). It is structurally similar to the GAF domains of cAMP- or cGMP-specific phosphodiesterases (PDE), including PDE10A^28^ and PDE2A^29^,^30^, with an RMSD of 2.5 Å over 130 Cα atoms and an RMSD 2.6 Å over 134 Cα atoms, respectively.

The Dcsbis molecules in the crystal are tightly dimerized by a four-helix bundle (α2, α5 of each protomer). In this structure, the α1 helix does not contribute to the dimerization (Fig. 2a), unlike the α1 helix of the GAF domain of PDE10A, which interacts with the α5 helix of the opposite molecule and plays a role in dimerization^28^. Hydrogen bonds and hydrophobic interactions are responsible for the majority of the interactions at the interface (Fig. 2c,d).

**Figure 4:**
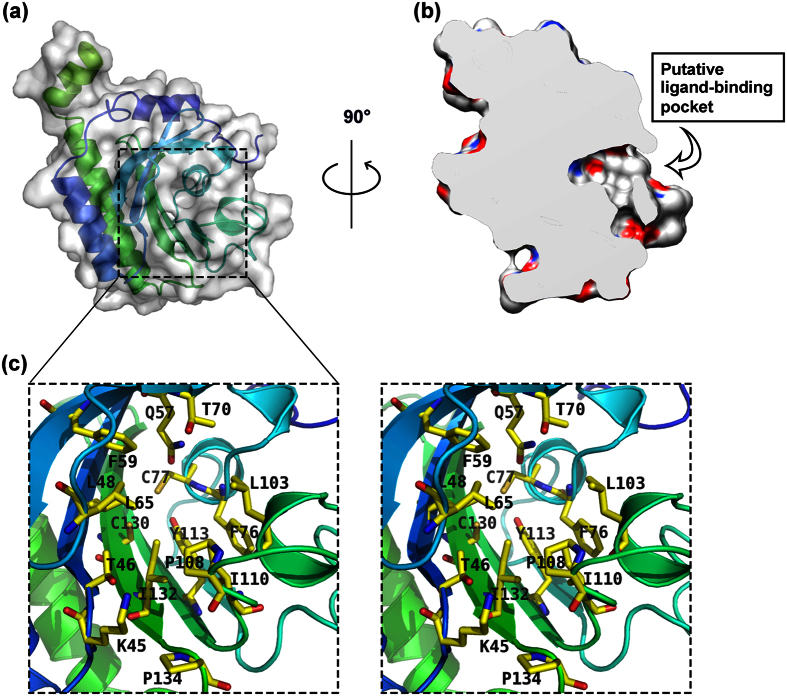
The putative ligand-binding pocket of the Dcsbis GAF domain. (**a,b**) Surface analysis of Dcsbis. The Dcsbis-GAF cartoon is colored in rainbow. (**c**) The stereo view of the residues that contribute to the formation of the putative ligand-binding pocket.

There is a large pocket located between the six-stranded β sheet floor and the roof made of α-helices α3 and α4 in the GAF domain (Fig. 4a–c). To investigate whether the GAF domain of Dcsbis can bind nucleotides, we performed an ITC analysis with cAMP and cGMP. A calorimetric analysis showed that Dcsbis did not bind cAMP or cGMP with any substantial affinity (Fig. S1). A sequence alignment of the Dcsbis GAF domain with the PDE GAF domains, the cyaB2 GAF domains and the PelD GAF domain is shown in Supplementary Fig. S2. Compared with some other GAF domains that bind nucleotides, the NKFDE motif, which is essential for nucleotide binding and the formation of the binding pocket^31^,^32^, is degenerate in the GAF domain of Dcsbis (Supplementary Fig. S2). This degeneracy may be the major reason that Dcsbis does not bind cAMP or cGMP. Thus, the GAF domain contains a large pocket that potentially binds to an unknown ligand.

Given the higher activity of full-length Dcsbis compared with that of the GGDEF domain alone (Fig. 1d) *in vitro*, it was speculated that the GAF domain promotes the DGC activity of the GGDEF domain. This increased activity may result from the dimerization via the GAF domain. The unknown ligand might play a role in the regulation *in vivo*.

### A self blocked I-site of Dcsbis

As shown in Fig. 1, Dcsbis exhibited much higher activity than the GGDEF domain alone, as well as the other well-studied DGCs. In addition, c-di-GMP affected the diguanylate cyclase activity of full-length Dcsbis. However, the effect of c-di-GMP on the catalytic activity of Dcsbis is significantly weaker than that on PleD and WspR^20^,^21^,^33^.

One hypothesis to explain this observation is that the product, c-di-GMP, does not bind as efficiently to full-length Dcsbis as it does to other proteins, reducing the product inhibition effects. To test this hypothesis, we measured the binding of c-di-GMP to Dcsbis with ITC, and no apparent interaction was detected (Supplementary Fig. S1), thus indicating that c-di-GMP does not readily bind to the I-site of full-length Dcsbis.

Further analysis of the crystal structures provided the molecular basis of the special property of the Dcsbis protein. Although the overall folding between the apo GGDEF domain in the full-length protein is very similar to that in the c-di-GMP bound state, the environment around the I-sites is very different. The superposition of the GGDEF domain/c-di-GMP complex onto the monomers of the Dcsbis full-length dimer revealed intermolecular interactions in the I-site. A protecting loop (Residue 120–125, between β5 and β6) from the GAF of one monomer (MonoA) inserts into the I-site of MonoB and clashes with the docked c-di-GMP molecule (Fig. 3c,d). Given that Dcsbis also exists as a dimer in solution, in the context of full-length Dcsbis, the I-site was mostly occupied by the neighboring molecule, which is buried inside the structure and is not accessible to c-di-GMPs. However, in the GGDEF domain alone, there is no protection from the GAF domain. The I-site is exposed to product as soon as the reaction starts. This finding may also explain the much lower activity of the GGDEF domain alone.

Thus, we propose that the protecting loop in the GAF domain prevents c-di-GMP from binding to the I-site (self-blocking I-site) of Dcsbis until c-di-GMP reaches a very high level in the reaction solution. This hypothesis is supported by the observation that the catalytic reaction turned off earlier when c-di-GMP was present, whereas the initial rate of the reaction was almost not affected (Fig. 1e). This hypothesis also fits with the observation that c-di-GMP only mildly inhibited DGC activity. The relief of product feedback inhibition by self-blocking of the I-site to maintain higher activity is a novel mode of DGC activity regulation.

### Enzymatic kinetics shows that c-di-GMP is not a noncompetitive inhibitor

**Figure 5:**
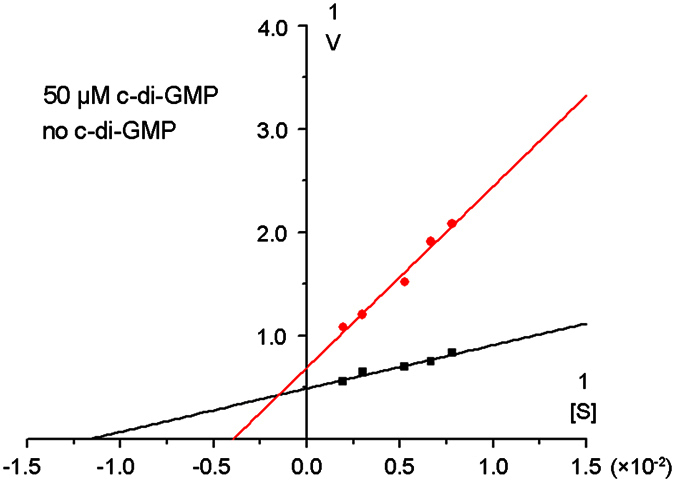
Double reciprocal plots of the kinetic study of Dcsbis protein in absent and present of c-di-GMP. This analysis was done by measuring three independent sets of experiments with 5 substrate concentrations (128 μM, 150 μM, 190 μM, 333 μM, 512 μM). The plots are generated by plotting 1/*V* as a function 1/[S]. The intercept on the vertical axis is 1/*V*_max_, and the intercept on the horizontal axis is −1/*K*_M_.

Previous studies on DGCs have proposed a widely accepted mechanism that c-di-GMP functions as an noncompetitive inhibitor that binds to the I-site of the canonical DGC domain, thus shutting down catalysis through an allosteric effect. Our structures and preliminary biochemical data, however, suggest a new mechanism for Dcsbis. Dicsbis has an intact I-site but is not inhibited by c-di-GMP as intensely as other canonical DGCs with an intact I-site. To clarify the inhibition mechanism of Dcsbic, we performed a kinetic study of catalysis with or without c-di-GMP in the reaction solutions. We hypothesized that if c-di-GMP is still a noncompetitive inhibitor for Dcsbis, the enzyme would show a much lower *Vmax* and an unchanged *Km* in the presence of c-di-GMP when the reaction begins. If this is true, then we would be able to conclude that c-di-GMP truly is a noncompetitive inhibitor of Dcsbis. Figure 5 shows the result of the kinetic study for Dcsbis. The enzyme had a *Km* of 88 μM and a *Vmax* of 2.1 μM/m without c-di-GMP. The enzyme had a much higher *Km* of 300 μM and a slightly lower *Vmax* of 1.6 μM/m when 50 μM of c-di-GMP was added prior to GTP. This result fundamentally deviates from the widely accepted theory of noncompetitive inhibition for c-di-GMP. The kinetics can be explained by a self-blocked I-site, which may be partially occupied at high c-di-GMP concentrations. The greatly increased *Km* suggests that c-di-GMP mainly binds to the active site as a competitive inhibitor, as in the case of XCC4471. Although our ITC data showed that c-di-GMP did not tightly bind to Dcsbis, the kinetic data suggest that c-di-GMP outcompetes GTP for the active site at comparable concentrations.

## Discussion

C-di-GMP is a ubiquitous signaling molecule in bacteria. The DGC and PDE proteins involved in the metabolism of c-di-GMP are redundant. A total of 39 proteins related to c-di-GMP homeostasis have been identified in *P. aeruginosa* PAO1 and PA14^22^. However, the biochemical characteristics and regulatory mechanisms of only a few of these proteins have been characterized. SadC, a GGDEF protein, has been reported to co-regulate biofilm formation and swarming motility^24^. PleD is a response regulator with two REC domains as well as a GGDEF domain that controls pole morphogenesis during cell differentiation^34^. WspR, which has a REC and a GGDEF domain, is the response regulator of a chemosensory system controlling biofilm formation^35^. Dcsbis is a diguanylate cyclase located on a genomic island^22^ that includes an N-terminal GAF domain fused in tandem to a GGDEF domain. Here, we report the function of the PA2771 protein. A ΔPA2771 mutant exhibited a phenotype different from those of well-studied GGDEF-containing proteins. It showed an increased swimming and swarming phenotype (Fig. 1a,B), indicating that the PA2771 gene should be related to pathways that regulate flagella function. However, the ΔPA2771 mutant had no apparent influence on biofilm formation (Fig. 1c). This result might be due to subcellular distribution of c-di-GMP^36^,^37^.[Fig f6]

Our studies showed that full-length Dcsbis possesses a high level of DGC activity, whereas its GGDEF domain has residual diguanylate cyclase activity. This result is similar to that of DgcA from *Rhodobacter sphaeroides*^38^ and MSDGC-1 from *Mycobacterium smegmatis*^39^. In both proteins, the DGC activity has been shown to largely decrease after the removal of the GAF domain. This finding suggests that the DGC activity of Dcsbis is modulated by the GAF domain. Although the GAF domain of Dcsbis is topologically similar to canonical GAF domains that bind to cNMP molecules, our calorimetric studies showed that Dcsbis binds neither cAMP nor cGMP with a meaningful affinity. However, a large pocket that might accommodate a ligand exists in the GAF domain. Thus, we suspect that an unknown ligand may bind to the GAF domain and somehow regulate the DGC activity.

From the crystal structure of full-length Dcsbis, we found that the GAF domain acts as a bridge in the dimerization of Dcsbis. This dimerization brings the GAF domain of one monomer close to the GGDEF domain of the other monomer. Furthermore, the I-site of the GGDEF domain from one monomer is blocked by the protecting loop between β5 and β6 of the neighboring GAF domain. The observation that c-di-GMP is not a noncompetitive inhibitor of full-length Dcsbis is supported by the crystal structure. The blockage of the I-site might be the primary cause of the high catalytic activity of Dcsbis and the loss of product inhibition. With this novel characteristic, Dcsbis is a highly efficient diguanylate cyclase that may be used in industrial applications to produce large amounts of c-di-GMP at a low cost.

**Figure 6 f6:**
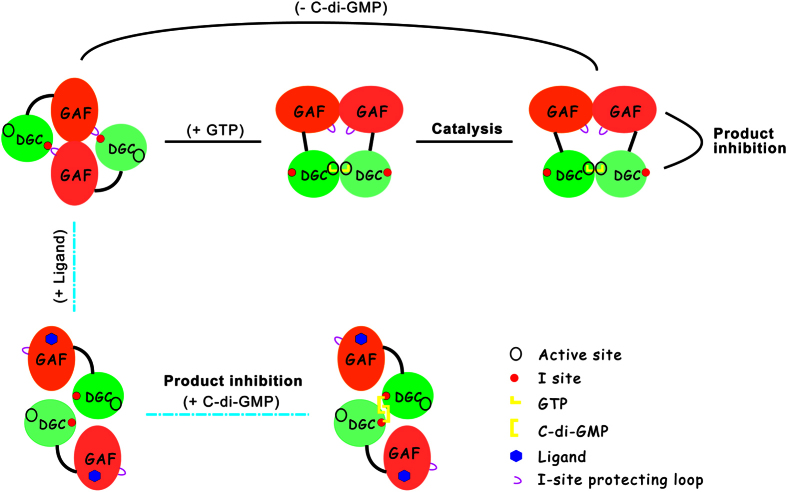
The proposed model for the activation and inhibition of Dcsbis. The model demonstrates the potential regulation mechanism of Dcsbis activity. In the absence of the regulatory ligand, substrate GTP, and product c-di-GMP, the Dcisbis protein dimerizes via the GAF domain, with the I-site blocked by the protecting loop (represented by the full-length Dcsbis structure), and thus is highly active; Upon binding to GTP, the active sites of Dcsbis dimer get close to each other and catalyze the formation of c-di-GMP; c-di-GMP induces slight product inhibition. Binding of the potential regulatory ligand to the GAF domain may induce conformational change in the GAF domain, and thus break the GAF dimerization and lead to the release of the blockage of the I-site. At this condition, the product c-di-GMP could bind to the I-sites of GGDEF domain and bridge the formation of a new dimer (represented by the structure of GGDEF domain/c-di-GMP complex).

From our structural and functional analyses, we propose a model for the activation and inhibition of Dcsbis (Fig. 6). The crystal structure of full-length Dcsbis showed that the inhibition site is buried inside and that the active site is exposed. This state might be one of the transition states during catalysis. We propose that when Dcsbis is active, as we assayed *in vitro*, GTP binds to the active site and induces a conformation change in the active dimer, thus leading to the proximity of the two active sites in both monomers. Two GTPs are then brought close to each other and form c-di-GMP. When the environment changes, bacteria favor a highly motive life style, and some unknown small molecules may bind to the GAF domain as a chemical signal. The binding of the signal molecule to the GAF domain would, in turn, trigger a significant conformation change and cause the protecting loop to leave the I-site. C-di-GMP in the cell would then bind to the exposed I-site, which would shut down the synthesis of c-di-GMP through an allosteric mechanism. Over time, the environment may change to favor lower motility, and the concentration of the signal molecule may drop, thus resulting in the release of a signal molecule from the GAF domain. The protecting loop would then block the I-site again and prevent the binding of c-di-GMP, which would turns on the high enzymatic activity once again. Because the peptide segment of the protecting loop also occurs in many Dcsbis homologues of *Pseudomonas* species, this mechanism may occur in many other bacteria (Fig. S3).

We demonstrated that Dcsbis is a functional diguanylate cyclase that tightly coordinates cell motility. However, the entire signaling pathway is largely unknown. Our results suggest that there should be an upstream signal molecule capable of binding to the GAF domain. The relevant downstream c-di-GMP receptor of the pathway is also unclear. We showed that the PA2771 gene is related to cell motility. Whether this gene is relevant to other cellular functions, such as drug resistance, virulence and cell division, remains an open question. All these issues are expected to be addressed through further research.

## Methods

### Bacterial strains and growth conditions

All relevant strains, plasmids and primers are listed in Supplementary Table S1. *P. aeruginosa* and *E. coli* strains were cultured in LB broth, except that the selective medium for *P. aeruginosa* was VBMM with citrate as a carbon source^40^. Antibiotic selection was performed with 100 μg/ml ampicillin and 10 μg/ml gentamicin for *E. coli* and with 300 μg/ml carbenicillin and 100 μg/ml gentamicin for *P. aeruginosa*.

### Mutant construction

An unmarked nonpolar deletion strategy was used to construct the ΔPA2771 mutant^41^,^42^. A 1.2-kb fragment was obtained by overlapping PCR and the ligation of a 0.6-kb upstream and a 0.6-kb downstream fragment relative to PA2771. The 1.2-kb fragment was subsequently cloned into the EcoRI and HindIII sites of pEX18Gm, thus generating the plasmid pKO2771. The plasmid pKO2771 was transferred to PAO1 through mating with *E. coli* DH5α cells containing the plasmid pRK2013 and *E. coli* DH5α cells containing PKO2771, thus generating a double-crossover recombinant. The ΔPA2771 mutant was isolated for gentamicin resistance and sucrose sensitivity.

The PA2771 gene was cloned into pUCP18 to generate the plasmid pPA2771, which was electroporated into WT PAO1 and the ΔPA2771 mutant, respectively.

### Motility and biofilm assays

LB medium with 0.3% (wt/vol) agar was used for the swimming motility assay. The bacteria from an overnight culture in LB agar (1.5%, wt/vol) were placed into swim plates with a sterile toothpick and incubated at 37 °C for 12 h. Swarming motility assays were performed in LB broth with 0.5% (wt/vol) agar.

Static biofilm assays were performed with 96-well microtiter plates^43^. Overnight cultures were diluted 1:100 into LBNS-medium. Then, 150 μl of each diluted culture were dispensed into wells and incubated for 24 h at 37 °C. The planktonic bacteria of each well were removed and washed. The biofilms were stained with a 1% crystal violet solution and measured spectrophotometrically at 560 nm.

### Protein expression and purification

The full-length and the GGDEF domain (residues 173-341) of the PA2771 gene were amplified from the genome of *P. aeruginosa* PAO1 and cloned into the pGL01 vector. *E. coli* BL21 (DE3) was used to express proteins. The transformed cells were grown at 37 °C until the absorbance (OD_600_) reached approximately 0.8, and this was followed by overnight induction with 0.2 mM isopropyl-β-D-thiogalactopyranoside (IPTG). Cells were harvested by centrifugation (4,200 rpm for 15 min at 4 °C); resuspended in lysis buffer (25 mM Tris-HCl pH 8.0, 200 mM NaCl), and lysed by sonication on ice. The soluble fraction containing Dcsbis was isolated from cell debris by ultra-centrifugation (14,000 rpm for 45 min at 4 °C) and subsequently purified to homogeneity by combining nickel affinity (Chelating Sepharose Fast Flow, GE Healthcare), ion exchange (Source 15Q HR 16/10, GE Healthcare), and size-exclusion chromatography (Superdex 200 10/300 GL, GE Healthcare) in 10 mM Tris-HCl pH 8.0, 100 mM NaCl, and 3 mM dithiothreitol.

The selenomethionine substituted protein was expressed in M9 medium with L-selenomethionine, and the purification procedure was the same as that of the native proteins. The pH of the medium was adjusted to 7.0 with 1 M Tris-HCl pH 8.0 (approximately 10 ml/L medium) before induction with IPTG.

### Crystallization, data collection and structure determination

Native Dcsbis crystals were grown in sitting drops at 20 °C by mixture of equal volumes of protein (7 mg/ml) with reservoir solution (10% PEG 8000, 0.1 M HEPES pH 7.5, 8% ethylene glycol). The selenomethionine-substituted protein was crystallized using a reservoir solution containing 10% PEG 10,000, 0.1 M HEPES pH 7.5 and 5% MPD. The crystals of Dcsbis (173-341) were obtained in a reservoir solution containing 0.5 M sodium citrate tribasic dehydrate and 0.1 M Tris-HCl pH 8.5. All diffraction data were collected at the Shanghai Synchrotron Radiation Facility (SSRF) beamline BL17U1 and processed with the software package HKL2000^44^. The selenium positions were identified using SHELXD^45^. Phase determination, density modification, and automated model building were performed using the program SOLVE/RESOLVE^46^,^47^. The atomic model was built using COOT^48^ and refined using PHENIX^49^. Then, the phase of the native full-length Dcsbis was obtained from molecular replacement with the Se-Dcsbis as a search model. The structure of Dcsbis (173-341) was determined by molecular replacement using the full-length Dcsbis structure as the search model. Refinement was performed in the same manner as that of the native full-length Dcsbis structure. The atomic coordinates and structure factors have been deposited in the Protein Data Bank under PDB ID codes 4ZMU (the full-length Dcsbis) and 4ZMM (the GGDEF domain of Dcsbis in complex with c-di-GMP).

### Diguanylate cyclase assays

A coupled spectrophotometric assay was performed to monitor the production of inorganic pyrophosphate (PPi) released from the cyclization reaction by using the EnzCheck Pyrophosphate Assay Kit (Invitrogen, E-6645)^46^. This kit includes the enzyme inorganic pyrophosphatase, which catalyzes the conversion of PPi present into two equivalents of Pi. In the presence of Pi and purine nucleoside phosphorylase (PNP), 2-amino-6-mercapto-7-methylpurine ribonucleoside (MESG) is converted into ribose 1-phosphate and 2-amino-6-mercapto-7-methyl-purine, thus resulting a shift in the absorbance maximum from 330 nm for the substrate to 355 nm. A kinetic analysis was performed by measuring three independent sets of experiments with 5 substrate concentrations. The double reciprocal plots were drawn using the Lineweaver–Burk equation by using the Origin software. The protein concentration was determined according to light absorption at 280 nm using a NanoDrop spectrophotometer.

## Additional Information

**Accession codes:** 4ZMM, 4ZMU.

**How to cite this article**: Chen, Y. *et al*. Dcsbis (PA2771) from *Pseudomonas aeruginosa* is a highly active diguanylate cyclase with unique activity regulation. *Sci. Rep*. **6**, 29499; doi: 10.1038/srep29499 (2016).

## Supplementary Material

Supplementary Information

## Figures and Tables

**Table 1:** X-Ray Data Collection and Refinement Statistics.

	SeMet-Dcsbis	Dcsbis	Dcsbis 173-341
**Data collection**			
Space group	*P*2_1_	*P*2_1_	*C*2
Cell dimensions			
*a*, *b*, *c* (Å)	74.428, 109.062, 112.765	73.609, 109.083, 111.850	93.275, 42.707, 106.372
α, β, γ (°)	90.00 95.450 90.00	90.00 95.88 90.00	90.00 106.75 90.00
Resolution (Å)	50.00–2.90	50.00–2.50	50.00–2.50
Measured reflections	297464	216967	46858
Unique reflections	40493	58687	13825
Completeness (%)	99.9 (99.8)*	96.6 (77.8)	98.2 (99.4)
Redundancy	7.3 (6.3)	3.7 (3.0)	3.4 (3.6)
*R*_*sym*_(%)^†^	8.7 (46.3)	6.9 (43.5)	4.7 (9.5)
*I*/(*I*)	21.69 (3.58)	19.86 (1.81)	46.34 (25.68)
**Refinement**			
Resolution (Å)		50.00–2.50	50.00–2.50
*R*_work_/*R*_free_ (%)		19.74/25.46	20.14/24.77
No. atoms			
Protein		10633	2513
Water		137	69
Ligand		–	92
*B*-factors			
Protein		55.57	44.91
Water		45.35	42.66
Ligand		–	31.46
RMSD			
Bond lengths (Å)		0.009	0.008
Bond angles (°)		1.246	1.317
Ramachandran plot (%)			
Favored region		95.5	97.2
Allowed region		4.5	2.8
Outlier region		0.1	0

*Values in parentheses are for reflections in the highest resolution shell.

^†^*R*_*sym*_ = Σ_*hkl*_Σ_*i*_|*I*(*hkl*)_*i*_ − <*I*(*hkl*)>|/ Σ_*hkl*_Σ_*i*_<*I*(*hkl*)_*i*_>, where <*I*(*hkl*)> is the mean intensity of multiply recorded reflections.
