# Supplementary material for: Dcsbis (PA2771) from Pseudomonas aeruginosa is a highly active diguanylate cyclase with unique activity regulation
Source: Sci Rep. 2016 Jul 8;6:29499. doi: 10.1038/srep29499 (PMC4937426; doi:10.1038/srep29499)
Supplement: Supplementary Information [file srep29499-s1.pdf]

Dcsbis (PA2771) from *Pseudomonas aeruginosa* is a highly active diguanylate cyclase with unique activity regulation

Ying Chen[1,4,5], Shiheng Liu[1,4] , Cuilan Liu[2,4], Yan Huang [1,4], Kaikai Chi[1], Tiantian Su[1], Deyu Zhu[1], Jin Peng[1], Zhijie Xia [3], Jing He[1], Sujuan Xu[1], Wei Hu* [1] and Lichuan Gu*[1]

[1]State Key Laboratory of Microbial Technology, School of Life Sciences, Shandong University, Jinan, Shandong, 250100, China
[2] College of Life Sciences, Shandong Normal University, Jinan, Shandong, 250014, China
[3]Institute for metabolic and neuropsychiatric disorders, Binzhou Medical University, Binzhou, Shandong, 256600, China

[4]These authors contributed equally to this work.
[5]Present address: Max Planck Institute for Developmental Biology, Dept of Biochemistry, Tübingen, Germany
*To whom correspondence should be addressed. Tel: (86) 531-88362039; Fax: (86) 531-88362039; E-mail: lcgu@sdu.edu.cn for LG and Tel: (86) 531-88364288; Fax: (86) 531-88364288; E-mail: hw_1@sdu.edu.cn for WH.

Supplementary table S1

Supplementary figures S1-S3

Table S1. Strains, plasmids, and primers used in the study

	Relevant genotype or sequence (5'-3')	Reference
***P. aeruginosa* strains**		
PAO1	Wide type	
PA2771 mutant	PAO1 Δ*PA2771*	This study
***E. coli* strains**		
DH5α	Wide type	
BL21(DE3)	Wide type	
**Plasmids**		
pEX18 Gm	Suicide cloning vector, oriT+, sacB+, Gm^r	26
pKO2771	*PA2771* knockout construct in pEX18, Gm^r	This study
pRK2013	Helper plasmids, ColE1, oriR, Tra, Mob, Km^r	53
pUCP18	*E. coli-P. aeruginosa* expression vector, Ap^r, Cb^r	54
pGL01	Expression vector, modified by pET15b, Ap^r	This study
pPA2771	*PA2771* in pUCP18, Ap^r, Cb^r	This study
pPA2771C	*PA2771*(residues 173-341) in pUCP18, Ap^r, Cb^r	This study
pPGL1	*PA2771* in pGL01, Ap^r	This study
pPGL173	*PA2771*(residues 173-341) in pGL01, Ap^r	This study
**Primers**		
KO2771UP5	CCGGAATTCGGGACTGTCGGCGAAT	
KO2771UP3	GGGCTGGAGGACTAGCCCTGGGAGCTCACTG GATCCTGATCACGGTGGAGA	
KO2771DN5	CCACCGTGATCAGGATCCAGTGAGCTCC CAGGGCTAGTCCTCCAGCCCTGC	
KO2771DN3	CCCAAGCTTCGATAGGCGGGCGCC	
KO2771S5	CGCATTCACCCAGATTGA	
KO2771S3	CCGAACACGATCACCTGCTGGATAA	
PA2771-*Bam*HI-F	ATAGGATCCATGCTCGCCTGCCCCCTC	
PA2771-*Xho*I-3T-R	ACGTCACTCGAGTCAGACGCTGACCACCCGG TT	
PA2771-173 -*Bam*HI-F	ATA GGA TCC GAG ATC GAC CGG GAG	
PA2771-341-*Hind*III-R	CCCAAGCTTTCAGACGCTGACCACCCGGTT	

Gm^r, gentamicin resistance; Tc^r , tetracycline resistance; Ap^r , ampicillin resistance; Cb^r , carbenicillin resistance; Km^r , kanamycin resistance.

References

1. Hoang TT, Karkhoff-Schweizer RR, Kutchma AJ, & Schweizer HP (1998) A broad-host-range Flp-FRT recombination system for site-specific excision of chromosomally-located DNA sequences: application for isolation of unmarked Pseudomonas aeruginosa mutants. (Translated from eng) *Gene* 212(1):77-86 (in eng).
2. Figurski DH & Helinski DR (1979) Replication of an origin-containing derivative of plasmid RK2 dependent on a plasmid function provided in trans. (Translated from eng) *Proc Natl Acad Sci U S A* 76(4):1648-1652 (in eng).
3. Yanisch-Perron C, Vieira J, & Messing J (1985) Improved M13 phage cloning vectors and host strains: nucleotide sequences of the M13mp18 and pUC19 vectors. (Translated from eng) *Gene* 33(1):103-119 (in eng).

Figure S1 Binding of cAMP, cGMP and c-di-GMP to Dcsbis analyzed by ITC.

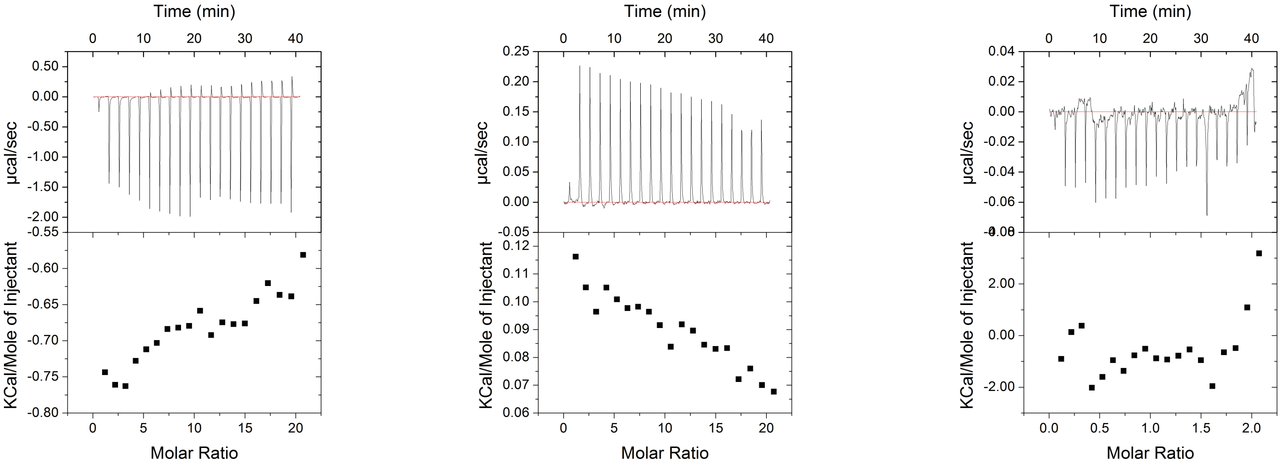

cAMP **cGMP** **c-di-GMP**

Figure S2 Multiple sequence alignment of the GAF domains from different proteins.

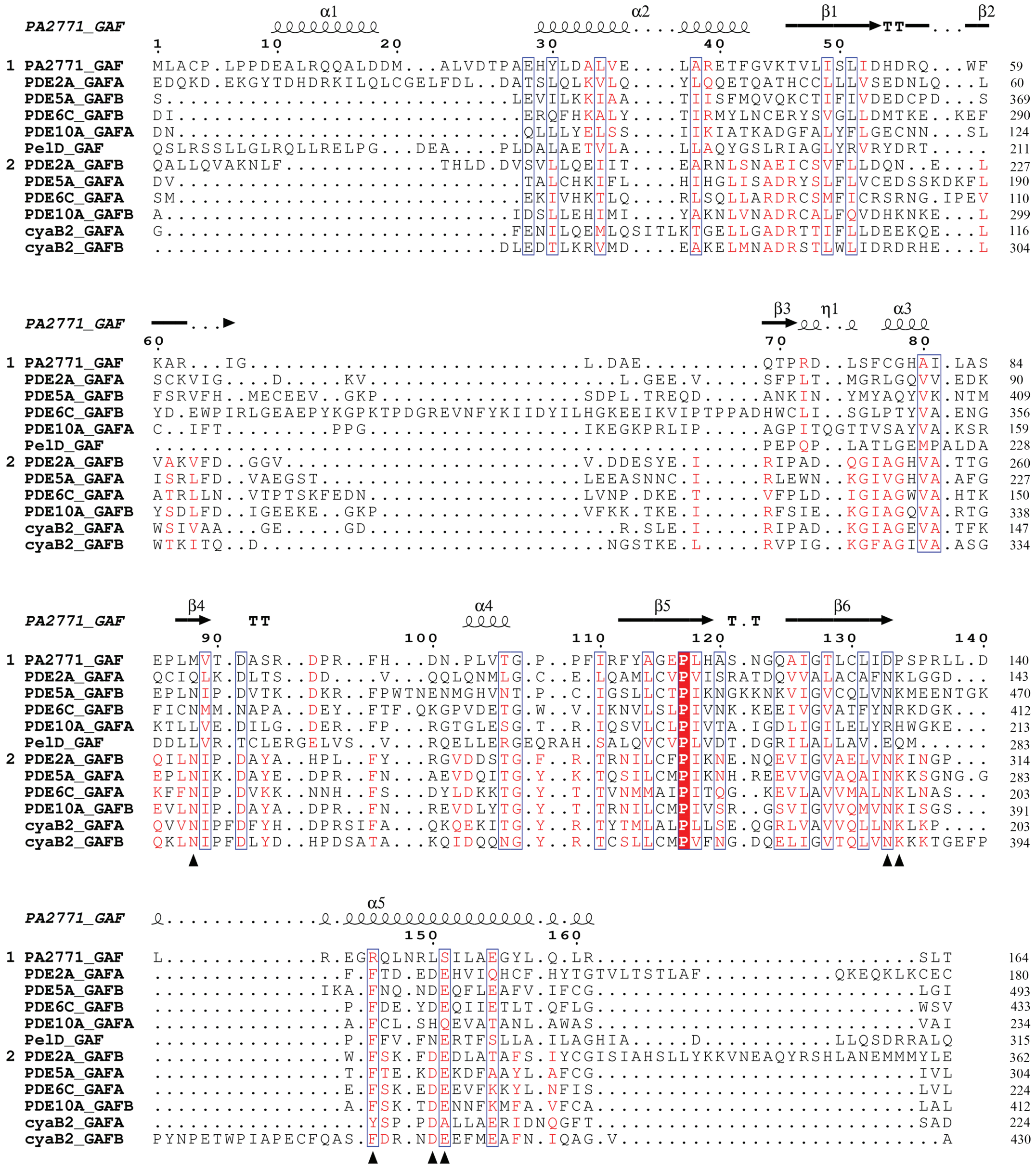

Figure S3
Sequence alignment of the protecting loop region through different Pseudomonas species

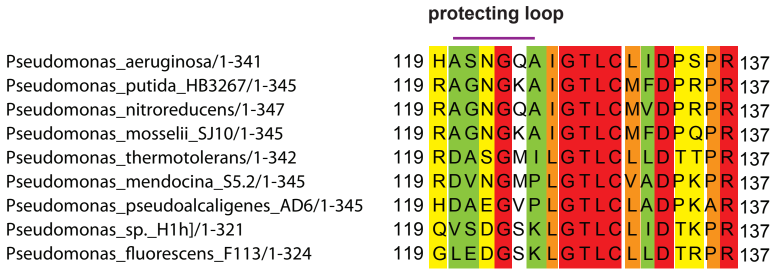

Alignment of the protecting loop through different Pseudomonas species

color code:
green: >40% conservation
yellow: >50% conservation
orange: >70% conservation
red: identical
